# Ultra-Short Laser Surface Properties Optimization of Biocompatibility Characteristics of 3D Poly-ε-Caprolactone and Hydroxyapatite Composite Scaffolds

**DOI:** 10.3390/ma14247513

**Published:** 2021-12-07

**Authors:** Albena Daskalova, Emil Filipov, Liliya Angelova, Radostin Stefanov, Dragomir Tatchev, Georgi Avdeev, Lamborghini Sotelo, Silke Christiansen, George Sarau, Gerd Leuchs, Ekaterina Iordanova, Ivan Buchvarov

**Affiliations:** 1 Institute of Electronics, Bulgarian Academy of Sciences, 72 Tzarigradsko Shousse Boulevard, 1784 Sofia, Bulgaria; emil.filipov95@gmail.com (E.F.); lily1986@abv.bg (L.A.); 2 Printivo Group JSC, 111 Tsarigradsko Shose Boulevard, 1784 Sofia, Bulgaria; r.stefanov@printivo.eu; 3 Institute of Physical Chemistry, Bulgarian Academy of Sciences, Acad. Georgi Bonchev Str. Bld. 11, 1113 Sofia, Bulgaria; dtachev@ipc.bas.bg (D.T.); g_avdeev@ipc.bas.bg (G.A.); 4 Institute for Nanotechnology and Correlative Microscopy GmbH (INAM), Äußere Nürnberger Straße 62, 91301 Forchheim, Germany; sotelo.lamborghini@gmail.com (L.S.); silke.christiansen@ikts.fraunhofer.de (S.C.); george.sarau@ikts.fraunhofer.de (G.S.); 5 Institute for Optics, Information and Photonics, Friedrich-Alexander-University, Erlangen-Nürnberg (FAU), Schloßplatz 4, 91054 Erlangen, Germany; gerd.leuchs@physik.uni-erlangen.de; 6 Max Planck Institute for the Science of Light, Staudtstraße 2, 91058 Erlangen, Germany; 7 Institute of Solid State Physics, Bulgarian Academy of Sciences, 72 Tzarigradsko Shousse Boulevard, 1784 Sofia, Bulgaria; ekiordanova@gmail.com; 8 Faculty of Physics, St. Kliment Ohridski University of Sofia, 5 James Bourchier Boulevard, 1164 Sofia, Bulgaria; ivan.buchvarov@phys.uni-sofia.bg

**Keywords:** ultra-short laser processing, bone tissue engineering, surface patterns, biodegradable polymers, antibacterial structuring

## Abstract

The use of laser processing for the creation of diverse morphological patterns onto the surface of polymer scaffolds represents a method for overcoming bacterial biofilm formation and inducing enhanced cellular dynamics. We have investigated the influence of ultra-short laser parameters on 3D-printed poly-ε-caprolactone (PCL) and poly-ε-caprolactone/hydroxyapatite (PCL/HA) scaffolds with the aim of creating submicron geometrical features to improve the matrix biocompatibility properties. Specifically, the present research was focused on monitoring the effect of the laser fluence (F) and the number of applied pulses (N) on the morphological, chemical and mechanical properties of the scaffolds. SEM analysis revealed that the femtosecond laser treatment of the scaffolds led to the formation of two distinct surface geometrical patterns, microchannels and single microprotrusions, without triggering collateral damage to the surrounding zones. We found that the microchannel structures favor the hydrophilicity properties. As demonstrated by the computer tomography results, surface roughness of the modified zones increases compared to the non-modified surface, without influencing the mechanical stability of the 3D matrices. The X-ray diffraction analysis confirmed that the laser structuring of the matrices did not lead to a change in the semi-crystalline phase of the PCL. The combinations of two types of geometrical designs—wood pile and snowflake—with laser-induced morphologies in the form of channels and columns are considered for optimizing the conditions for establishing an ideal scaffold, namely, precise dimensional form, mechanical stability, improved cytocompatibility and antibacterial behavior.

## 1. Introduction

Different types of scaffolds are required for insertion in the place of body injuries in order to facilitate tissue repair. Despite the huge impact that medical implants have on patients’ health and quality of life, the possibility of side effects after implantation remains. One of the most common complications is the development of a bacterial infection associated with the implant itself. *S. aureus* and *S. epidermidis* have been listed as the main pathogens that colonize implants and lead to severe inflammation [1]. When the implant is inserted at the site of injury, both human and bacterial cells compete for a place to attach to the material’s surface. The initial adsorption of bacter ia onto an inert and abiotic implant is unspecific and directed mainly by electrostatic, hydrophobic and van der Waals forces [2]. After they are attracted to the implant, microbes could bind irreversibly to the surface itself or to various serum proteins that have already been randomly adsorbed to the implant surface. This specific irreversible binding occurs via adhesin molecules that are part of the outer bacterial membrane [3,4]. Once the pathogens have attached to the surface, they proliferate and interact with each other in order to form an extracellular polysaccharide matrix—the bacterial biofilm [5]. The microbes, being embedded in such a layer, could become resistant to common antibiotic treatments. Consequently, the task of eliminating the developed infection becomes extremely hard. This layer allows the accumulation of bacteria that can be released over time, causing chronic inflammation, and hence, increasing the possibility of implant rejection by the body [6]. The bacteria–surface interaction is affected by different properties and characteristics of the material such as the surface chemistry and energy, the level of hydrophobicity, the topography, morphology and roughness [5,6,7,8,9].

The effects of topographical features and roughness profile of different surfaces on bacterial attachment have been widely explored [10,11,12]. Generally, with the increase in surface roughness, the surface area suitable for microbial adhesion also increases, which, in turn, results in a higher chance of a biofilm formation [6,13]. Despite this general observation, it has also been discovered that surfaces with higher roughness allow the adsorption of serum proteins. In consequence, a thin coating is formed which masks the rough nature of the surface and impairs the interplay between the bacteria and the material [14]. The surface topography and morphology are two key factors that influence bacterial behavior and are not strongly correlated to the surface roughness—different morphologies could show very close roughness values (expressed as root mean square (RMS) or arithmetic average (R_a_)) but could have opposite effects on bacterial or human cells [15,16]. Topographical features on a micro- or nanoscale could impact bacterial attachment via hydrodynamic interactions or by physicochemical forces and chemical gradients, respectively [17]. Various hierarchical patterns with pre-defined dimensions on both a micro- and nanoscale have been explored as ways to develop antimicrobial surfaces [18,19,20,21]. Surface features with different geometries such as pillars, single pits or channels could be realized via lithographic methods, etching or laser surface processing [20,21,22].

A major concept behind the use of lasers for material processing is to achieve an improved and localized modification process as well as to deliver the pulse energy in a more defined and effective manner [23]. Laser ablation with ultra-short laser pulses has been widely explored as a method to obtain defined, clean and precise superficial modification with various applications. The duration of the pulse determines the mechanisms via which the light energy propagates through matter and affects the material [24]. Upon the initial interaction between the laser pulse and the material, the time that is required for the pulse energy to be conveyed to the structural lattice of the molecules comprising the material is approximately 10 picoseconds [23]. Thus, in the case of nanosecond laser pulses, the interval between two consecutive pulses is long enough for the thermal energy to build up and create a heat-affected zone beyond the borders of the laser-affected zone itself. Within this zone, the lattice of the molecules is heated, and uncontrollable melting and evaporation of material is noted [25]. On the contrary, in the case of ultra-short laser pulses, no heat conduction to the structural lattice is observed; hence, no undesired damage to the surrounding material is detected. The femtosecond laser material processing leads to clean and precise texturing of the material’s surface without significant expulsion of melted material in the surrounding zones that could otherwise obliterate the obtained patterns [25]. Different surface modifications result in a change not only in the morphological features of the material, but also in an alteration of the surface physicochemical properties. For example, wettability, defined by the surface energy and roughness, is a key property of materials that can affect the attachment of microbes or various molecules to a surface [6]. By applying femtosecond laser pulses to influence surface topography, the degree of wettability can be finely tuned from hydrophobic to hydrophilic, or vice versa. The laser treatment induces changes in the contact surface area and in the chemical groups exposed on the surface [26]. Furthermore, in light of the development of antimicrobial surfaces, ultra-short laser processing of materials represents a technique that is contactless and does not require any additional chemical or biological treatments. Moreover, the possibility of material processing at a micro- or nanoscale leads to the formation of highly precise surface patterning with strict control over both the dimensions of the morphological features and the physiochemical properties of the processed material.

Since orthopedics is the field in which medical implants are utilized most often, there is a need for the development of novel materials and surfaces that could repel potential bacterial adhesion, stimulating at the same time their own integration within the human body. The gold standard materials for prosthetics (e.g., titanium and its alloys) have proven advantages; however, as mentioned earlier, they are bioinert and allow for unspecific adsorption of bacteria, which could lead to the development of a microbial biofilm [27]. Various materials of both natural and synthetic origin have been under thorough investigation as alternatives to metallic implants. This paper focuses on the application of the synthetic polymer poly-ε-caprolactone (PCL). This semi-crystalline thermoplastic polymer has a melting point of 58–63 °C and a glass transition temperature of 60 °C. The combination of non-polar methylene and polar ester groups that build up the structure of PCL determines the biodegradability of this material [28]. Due to the presence of a larger number of hydrophobic –CH_2_ groups within its structure, the full hydrolysis of the material could take up to 4 years. Apart from its good biodegradability, PCL also shows great biocompatibility as no immune reaction is triggered, neither soon after implantation in the body nor during hydrolysis [29]. In addition, since the material preserves its semicrystalline structure at physiological conditions, its overall mechanical integrity is not compromised. The natural toughness and elasticity are maintained for a long period after implantation [28]. These properties make the PCL a very prominent material for bone tissue engineering. PCL material demonstrates hydrophobic properties which have been investigated to reduce the attachment of *S. aureus* RP62A strain, the most common pathogen in healthcare-associated infections [30]. Generally, the variation in proteins and other macromolecules within the cell walls of the bacteria changes the bacterial affinity towards hydrophobic and hydrophilic surfaces [31]. The inherent hydrophobicity of PCL could be beneficial for antimicrobial studies; however, it has been shown that osteoblasts adhere and differentiate better on hydrophilic surfaces [32]. For improved integration of the material in the body, it is important to reduce the level of hydrophobicity of the created 3D PCL scaffolds. There are various treatments that could achieve this goal, such as coating of the material with proteins such as fibronectin (part of the native extracellular matrix (ECM)), growth factors or with minerals such as hydroxyapatite (particularly for bone tissue engineering) [28]. These coatings induce faster and more efficient initial attachment, adhesion and proliferation of the cells since they mimic the native extracellular environment more closely. As mentioned above, surface roughness has an important role in the physicochemical properties of the material; however, it also has influence on cell activity and cell orientation [33]. Bone matrix, on the other hand, is composed mainly of calcium phosphate (around 60%) in the form of hydroxyapatite (HA) [34]. It is the prime component of the implant’s “binding material” with the surrounding tissues, making a mechanically stable connection with them [35]. As a result, HA incorporation into the 3D-printed PCL scaffolds could enhance their recipient implantation.

In this work, we aimed at developing patterned surfaces that could stimulate bone tissue regeneration and impair bacterial attachment and subsequent biofilm formation. For achieving this goal, we applied femtosecond (fs) laser treatment on 3D-printed PCL and poly-ε-caprolactone/hydroxyapatite (PCL/HA) scaffolds with a pre-defined pore geometry. Processing the surfaces of both types of matrices resulted in the development of distinct patterned topography represented by either parallel microchannels or single microprotrusions. Additionally, the formation of microchannels on the PCL scaffold led to a sharp increase in the degree of wettability as complete wetting of the surface was observed. Based on our findings, we hypothesize that depending on the obtained modification, the PCL matrices could be used for stimulation of the bone tissue regeneration (microchannels) or bacterial entrapment and biofilm disruption (microprotrusions).

## 2. Materials and Methods

### 2.1. Fabrication of 3D-Printed PCL and PCL/HA Scaffolds

**Figure 1:**
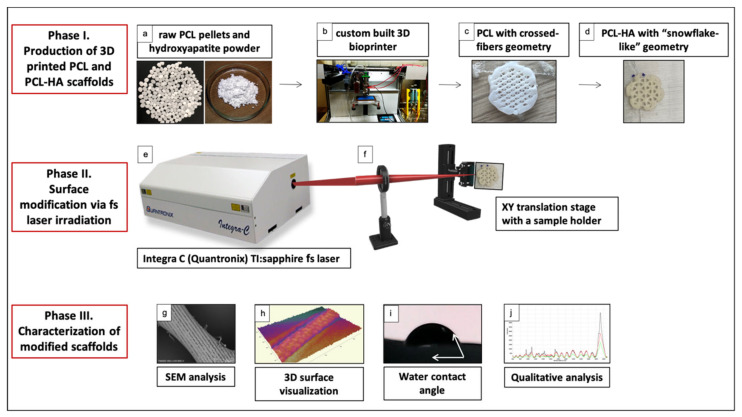
A visual representation of the experimental procedure described in this paper. (**a**–**d**) The first phase of the experimental work, involving the fabrication of 3D-printed PCL and PCL/HA scaffolds with two different geometries; (**e**,**f**) surface processing of the fabricated materials with a femtosecond laser; (**g**–**j**) the last phase comprising physical and chemical characterization of the processed surfaces.

For the fabrication of pure PCL scaffolds, PCL pellets (Figure 1a) (average Mn: 45,000; Sigma-Aldrich, St. Louis, MO, USA) were heated up to 110 °C. PCL/HA scaffolds were prepared following an established protocol with additional modifications [36]. Briefly, PCL pellets were melted at 350 °C in order to incorporate hydroxyapatite (powder; Sigma-Aldrich) completely and to obtain a homogeneous viscous mixture (manual stirring). The two materials were mixed in a ratio of 70:30 w/w % (PCL:HA). This weight ratio was selected based on the results from the research of Zheng et al. [34]. The composite was transferred to the cartridge of a custom-built 3D printer (Figure 1b), utilizing a pneumatic extrusion system. Scaffolds were printed through a stainless steel luer lock nozzle tip with a 280 μm inner diameter at a temperature of 110 °C, printing speed of 100 mm/min, 0.3 mm layer height and 50–70% infill. The printing followed two different geometries: crossed fibers and “snowflake”, based on literature research (Figure 1c,d) (discussed in detail in Section 4). The average thicknesses of the fibers of the PCL and PCL/HA scaffolds were 390 μm and 563.9 μm, respectively. A separate sample (solid plate, 2 × 2 cm) was 3D printed and further used for water contact angle and microhardness measurements.

### 2.2. Irradiation of PCL and PCL-HA Scaffolds with Femtosecond Laser Pulses

The femtosecond (fs) laser setup includes a Ti:sapphire mode-locked laser (Quantronix-Integra-C System, Hamden, CT, USA) (Figure 1e) with a pulse duration of 130 fs, a repetition rate of 1 kHz and a central wavelength λ = 800 nm. The repetition rate was reduced to 500 Hz for all experiments by an optical chopper. The scaffolds were positioned on a vertical XY translation stage (perpendicular to the direction of the laser beam) (Figure 1f). The number of pulses (N) delivered at each laser spot was defined by the speed of the translation stage, controlled by LabView software. The number of applied laser pulses (N) was varied between 2 and 10 and the laser fluence (F) between 0.04 and 0.08 J/cm^2^ in order to choose the best conditions for achieving column- and channel-like structures. The application of diverse numbers of Ns was performed to estimate the best condition for formation of the abovementioned structures. The selection of N = 2 and N = 10 was performed in relation to the morphological analysis. It was found that the application of a lower number of Ns triggered elevation of material and development of bump-like structures simulating column form. By further increasing N, we observed a transition of the structures’ shape to channel-like morphology; this transition was explicitly monitored for the application of N = 10. This led to the conclusion that the number of applied laser pulses significantly affects the final shape of the obtained structures. Two additional parameters that were taken into consideration were the distance between each two consecutive laser-created spots (d_x_) and the distance between two separate rows (d_y_), which varied between 32 µm and 45 µm.

### 2.3. Characterization of Laser-Irradiated PCL and PCL/HA Scaffolds

#### 2.3.1. Scanning Electron Microscopy and Analysis of Elemental Composition via Energy-Dispersive X-ray Spectroscopy (EDX)

The irradiated PCL samples were analyzed via scanning electron microscopy (TM4000 Hitachi High-Tech Europe; JEOL JSM 5510; “Lyra”, Tescan Orsay Holding). Prior to scanning, the samples were sputter-coated with a thin layer of gold (approximately 10 nm thick) to increase their conductivity. To the samples analyzed via the setup provided by Tescan (Brno, Czech Republic), carbon thread coating (6–10 nm thick) was applied. Images were obtained at 10 kV (JEOL JSM 5510, JEOL, Tokyo, Japan), 15 kV (TM4000, Hitachi High-Tech Europe, Krefeld, Germany) and at 5 kV (“Lyra”, Tescan, Brno, Czech Republic).

The analysis was performed by SEM-TESCAN/Lyra equipped with an EDX module (Quantax 200, Bruker, Karlsruhe, Germany). Prior to observation, the scaffolds were coated with a thin layer of carbon. Three spots per area (laser-treated and untreated) were chosen for the analysis and data were obtained at an operational voltage of 20 keV.

#### 2.3.2. Analysis of Surface Modifications via Optical Profilometer

The femtosecond-modified surfaces of the samples were further observed and evaluated via an optical profilometer (Zeta-20, Zeta Instruments, Gallatin Valley, MT, USA). The setup performed a vertical scan over a specific area on the sample, yielding 2D and 3D images in true colors. By utilizing the Zeta Optics Module, not only the XY location of a particular point of interest was recorded, but also the exact height for every Z position was measured. The vertical resolution (Z) was less than 1 nm, while the field of view for the images acquired was 476 μm × 357 μm. The magnification range of the profilometer was 5–100× as all images were obtained at 20×. The visualization of the surface of the scanned areas in true colors allowed measurements of surface roughness, for which the root mean square (RMS; every height value within the dataset is squared and the square root of the mean is then used) values were evaluated. The RMS values were averaged on three roughness assessments over each area of interest. All images obtained from the optical profilometer were analyzed via ProfilmOnline (www.profilmonline.com, accessed on 20 October 2021).

### 2.4. Computed X-ray Tomography for Evaluation of Surface Roughness after Femtosecond Laser Ablation

The optical tomography analytical method allows 3D visualization of an object. For this purpose, X-rays pass through the specimen, and by becoming attenuated, a series of 2D images are acquired. A 3D image allowing every possible cross-section and volume and surface morphometric analysis is obtained by computed reconstruction. Tomographic scanning was performing with Bruker SkyScan 1272 (Bruker, Berlin, Germany) under the following conditions: X-ray tube voltage, 70 kV; current, 142 µA; pixel size, 3 µm; optical magnification, 2.4. The reconstruction was performed with the InstaRecon software, but further analysis was performed by Bruker’s dedicated software CTAn, CTvox and DataViewer.

### 2.5. Raman Spectroscopy and X-ray Diffraction Analysis (XRD) for Assessment of Changes in Structural Conformation of PCL and PCL-HA

Raman spectroscopy is used for the characterization of molecules’ structure. The technique relies on the inelastic scattering of light by matter. The analysis was performed by LabRAM HR Evolution (Horiba, Kyoto, Japan) with a 10× objective. All samples were measured using a 532 nm laser with a power of 1.68 mW. The results were analyzed via Spectragryph software and all plots were baseline-corrected.

X-ray crystallography results were obtained via Philips PW1050 X-ray diffractometer system (Philips, Amsterdam, The Netherlands) equipped with a copper anode and a secondary monochromator of the diffraction beam. The measurements were taken within the range of 5–85° θ2 with a step size of 0.05° θ2 and 3 s time of exposition. The phase identification was acquired via QualX2 software and Crystallography Open Database.

### 2.6. Wettability Assessment of PCL and PCL/HA Scaffolds via Water Contact Angle (WCA) Measurement

The WCA measurements were performed by pipetting a drop of deionized water with a volume of 1 µL onto the laser-treated and the control areas on the 3D-printed solid block of PCL. The changes in the contact angle values were monitored for 10 s after the initial deployment of the drop onto the scaffold. The experiment was replicated 3 times per modified zone. The WCA was measured by ImageJ software (ImageJ open software, downloaded from imagej.nih.gov), using a contact angle plug-in (downloaded from imagej.nih.gov, accessed on 1 September 2021). Two-way ANOVA analysis was applied to assess the statistical significance of the results. Additionally, the wettability assessment was also performed on the scaffolds with the two types of geometries. Since both types of geometries used for the scaffold production lead to the formation of voids (pores) between fibers, the drops were deposited on 3 different points within the modified area. The average value was used. The scaffolds with “snowflake” geometry had larger voids; thus, the points for drops were chosen to be within an area where the fibers cross each other.

### 2.7. Microhardness Test of PCL and PCL/HA 3D Scaffold

Digital Display Micro Vickers Hardness Tester HVS-1000 (Shandong Liangong Group, Shandong, China) equipped with an optical CCD camera and an image processing system was used for samples’ Vickers microhardness evaluation. The tests were performed at 400× optical magnification in testing regime and test force of 2.94 N/0.3 kg. Each obtained value was averaged over 5 separate measurements performed on control and fs-treated PCL and PCL/HA blocks and 3D samples (see paragraph 3.5). Tukey’s multiple comparisons test was applied to investigate the relationship between the separate groups.

## 3. Results

### 3.1. Morphological Studies of PCL and PCL/HA Matrices

**Figure 2:**
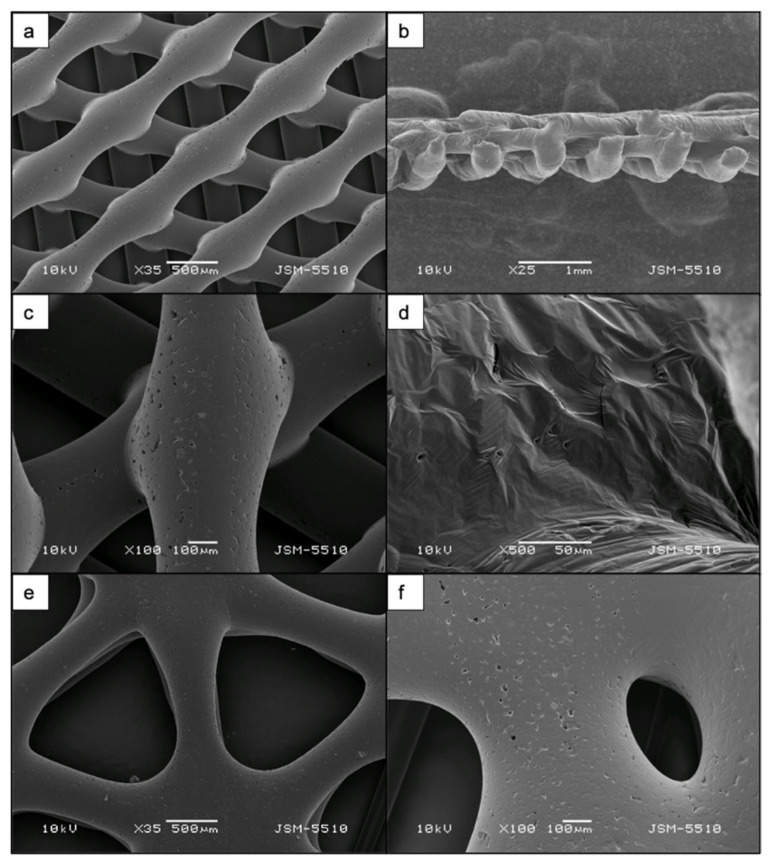
SEM images of untreated 3D-printed PCL scaffold. (**a**) The scaffold was printed with a specific pre-defined geometry type wood pile. (**b**) A cross-section of the scaffold. (**c**,**d**) Insights on the inherent porosity. (**e**) Snowflake-like geometry of PCL scaffold with larger pores between fibers. (**f**) A magnified image showing the inherent porosity of the snowflake-like scaffold.

Prior to laser ablation, the structure and the surface of the matrices were analyzed via SEM (Figure 2). Images of scaffold cross-section with crossed-fibers geometry showed that the integrity of the single fibers was preserved after printing without detection of collapse between the separate fibers. Additionally, the analysis revealed details on the inherent porosity of the material, which is of key importance when tests with human cells are performed. The average diameter of the pores was 11 μm. Concerning the two types of geometries chosen for the printing process, both lead to the formation of triangular pores in between the separate fibers. The average diameter of pores of the wood pile and the snowflake-like scaffold was 700 μm and 800 μm, respectively (discussed in detail in Section 4).

**Figure 3:**
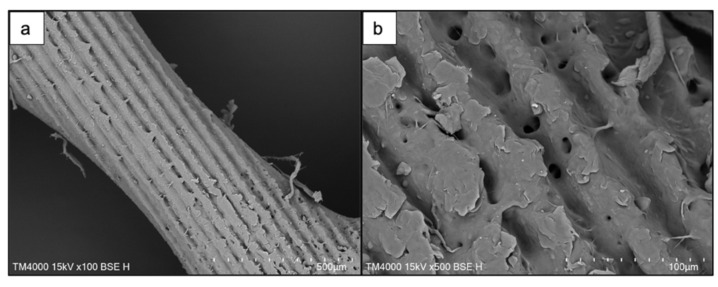
Formation of microchannels on a PCL scaffold after irradiation with a femtosecond laser. (**a**,**b**) Laser-produced parallel channels on scaffold surfaces. Parameters of laser processing: λ = 800 nm; F = 0.08 J/cm^2^; N = 10.

**Figure 4:**
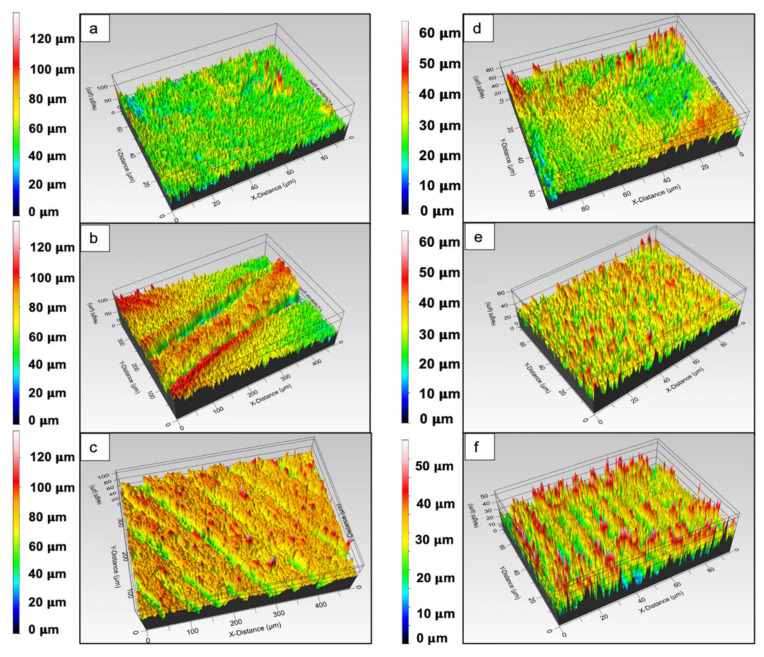
The 3D profiles of PCL and PCL/HA scaffolds treated with a femtosecond laser. (**a**) Control PCL scaffold with RMS: 8.98 μm; (**b**) modification on a single fiber, corresponding to Figure 6d (λ = 800 nm; F = 0.08 J/cm^2^; N = 2) with RMS: 6.7 μm; (**c**) PCL scaffold, corresponding to Figure 3b (λ = 800 nm; F = 0.08 J/cm^2^; N = 10) with RMS: 8.1 μm; (**d**) PCL/HA scaffold with RMS: 4.48 μm; (**e**) PCL/HA scaffold, corresponding to Figure 7b with RMS: 6.77 μm; (**f**) PCL/HA scaffold, corresponding to Figure 5b with RMS: 6.68 μm.

**Figure 5:**
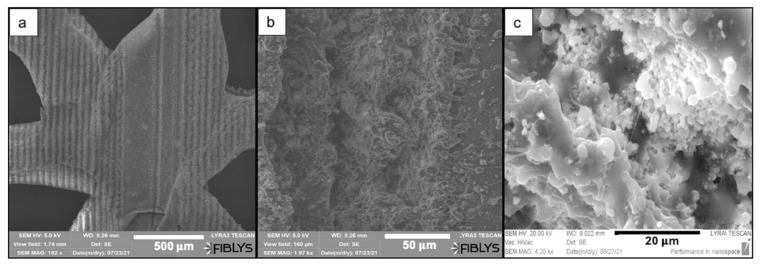
Creation of microchannels on a PCL/HA scaffold via femtosecond laser irradiation. (**a**) a front view of the fs laser-processed scaffold (λ = 800 nm; F = 0.08 J/cm^2^; N = 10); (**b**) a magnified image of the formed parallel channels; (**c**) details on the morphology of the inner side of the microchannels.

SEM images revealed the effects of the laser treatment on the surface morphology of both PCL and PCL/HA scaffolds. After preliminary tests with femtosecond laser pulses, the optimal laser parameters that were chosen for further experiments were as follows: λ = 800 nm; F = 0.08 J/cm^2^; N = 2 or 10. Application of a higher number of laser pulses per spot (N = 10) to the surface of the scaffold led to the formation of microchannels with an average diameter of 25 μm on PCL scaffolds (Figure 3 and Figure 4) and 43.2 μm on PCL/HA scaffolds (Figure 4 and Figure 5).

The roughness within separate channels was measured as RMS values and ranged between 1.2 μm and 5.3 μm. Overall, it was observed that the laser irradiation improved the inherent porosity of the PCL matrices (Figure 3b), particularly within the channels. Channels, which do not appear smooth and flat, but are rather rough and porous, are expected to have a positive impact on cell attachment and guidance [37,38,39]. Furthermore, both numbers of applied laser pulses did not lead to any significant unwanted collateral damages of the material that could possibly affect the obtained surface modifications. However, “bridging” was observed between separate channels, formed by melted material fusion. These irregularities are monitored on SEM images (Figure 3) and on the 3D true-color visualization obtained by the optical profilometer (Figure 4b) of the PCL matrix.

Comparing the PCL and the PCL/HA scaffolds, it could be easily seen that the microchannels obtained on the composite showed less porosity; however, a larger amount of melted material remained within the channel, contributing to their roughness and more defined borders (Figure 4f and Figure 5c).

**Figure 6:**
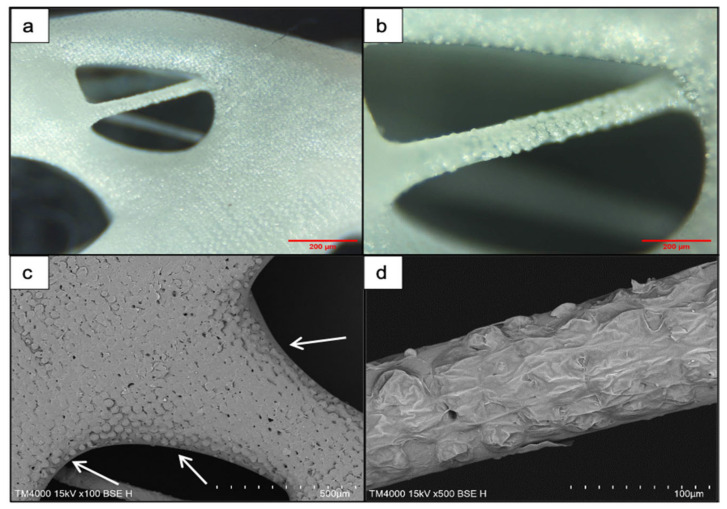
SEM images of microcolumns on 3D-printed PCL scaffolds obtained with laser parameters: λ = 800 nm; F = 0.08 J/cm^2^; N = 2. (**a**,**b**) Light microscopy images of the superficial laser modifications. (**c**,**d**) SEM images of the same modifications (white arrows point at single columns formed at the edges of the pores).

**Figure 7:**
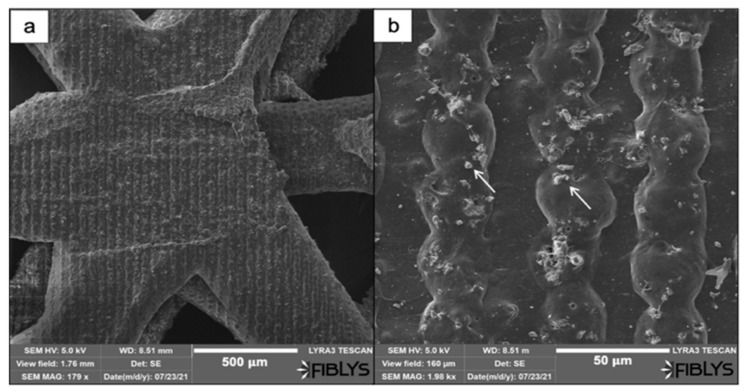
Formation of 3D protrusions on a PCL/HA scaffold after femtosecond laser ablation. (**a**) Front view of an fs-modified matrix (λ = 800 nm; F = 0.08 J/cm^2^; N = 2); (**b**) a magnified image of arrays of microprotrusions with an average diameter of 32.5 μm (white arrows indicate the presence of hydroxyapatite crystals, exposed on the surface of the protrusions).

Decreasing the number of applied laser pulses from 10 to 2 led to the formation of microcolumns onto the surface of both scaffolds (Figure 6 and Figure 7). On the pure PCL scaffold, more clear and separate columns were observed towards the edges of the scaffold, near the larger pores between fibers (Figure 6c). The columns that formed in the inner area, where fibers merge into each other, formed a continuous layer of melted material. The range of distance between separate microcolumns was measured, 4–13 μm, as well as their average diameter, 25 μm. The distance between the columns should be reduced as the initial spacing was too large for a single bacterium (known to range between 0.5 and 2 μm in size) and could possibly allow the agglomeration of microbes [40].

When the same laser parameters were used for the modification of PCL/HA scaffolds (λ = 800 nm; F = 0.08 J/cm^2^; N = 2), the formation of microprotrusions was monitored. In this case, they appeared as separate parallel arrays (Figure 7). The average diameter of the protrusions was estimated to be 32.5 μm. The laser irradiation did not lead to the formation of a melted layer, but only to the partial collapse of the microprotrusions. On top of the 3D formations, as a result of the interaction between the laser beam and the material, hydroxyapatite crystals were elevated (Figure 7b).

The presence of the elements was confirmed by an EDX analysis, which also provided an overall quantitative analysis concerning the elements composing the scaffold (Table 1).

Comparison between control and laser-treated areas (λ = 800 nm; F = 0.04 J/cm^2^; N = 10) revealed higher weight % of calcium (Ca) and phosphorus (P) elements after fs laser processing.

**Figure 8:**
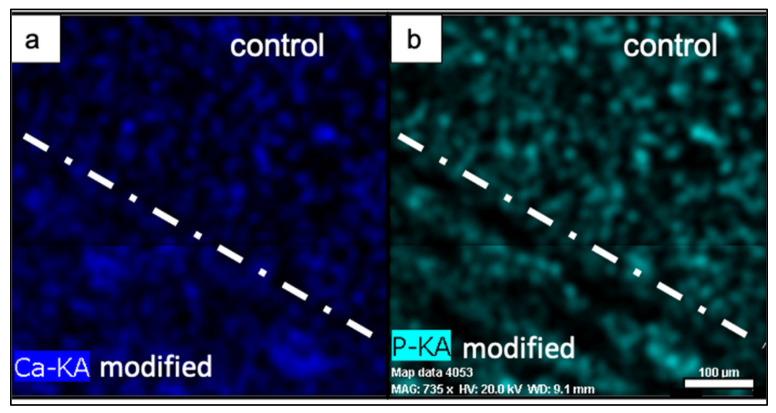
Mapping of Ca (**a**) and P (**b**) elemental signal distribution on modified and control surfaces of PCL/HA scaffold (λ = 800 nm; F = 0.04 J/cm^2^; N = 10). Scale bar: 100 μm for both (**a**) and (**b**).

For a better investigation of the distribution of Ca and P elements within the composite scaffold, an EDX mapping was also performed (Figure 8). Bright and homogeneous signals of both elements were detected from both laser-treated and untreated areas, indicating the even distribution of hydroxyapatite within the composite scaffold. The femtosecond laser irradiation did not lead to the removal of elements within the modified areas, but rather to their spatial rearrangement and clustering.

As a basic tendency, it can be summarized that the differences in the obtained morphology after laser irradiation between PCL and PCL/HA scaffolds could be attributed to the presence of hydroxyapatite and its interaction with the femtosecond laser radiation.

### 3.2. Surface Roughness Assessment of Femtosecond Laser-Processed Scaffolds

**Figure 9:**
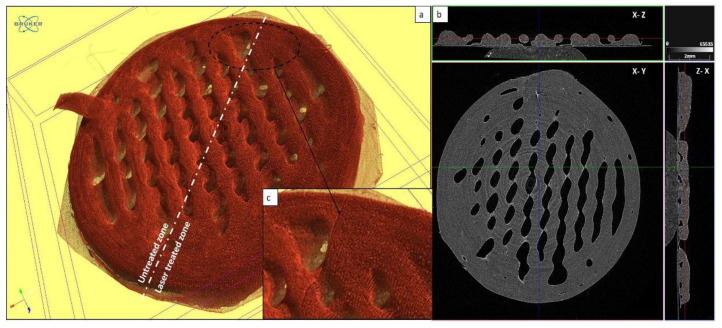
Visualization of laser-ablated PCL scaffolds by computer tomography; (**a**) 3D image of the scaffold treated with a femtosecond laser in half (white axis divides untreated and laser-treated areas) (λ = 800 nm; F = 0.08 J/cm^2^; N = 10); (**b**) the scaffold presented from different planes; (**c**) magnification image of the limit between the control and laser-processed zone.

In order to further investigate the effects of femtosecond laser treatment, the surfaces of both modified and unmodified parts of the scaffolds were visualized via computer tomography (Figure 9).

**Figure 10:**
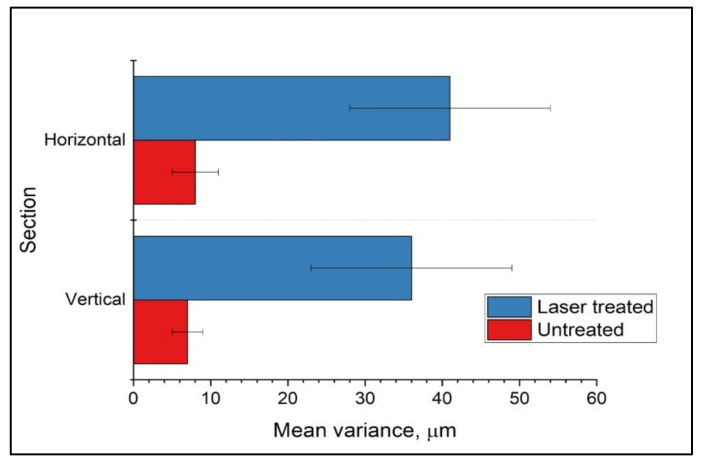
Comparison of surface roughness between laser-ablated (in blue) (λ = 800 nm; F = 0.08 J/cm^2^; N = 10) and untreated areas (in red) on the PCL scaffold.

The surface roughness of the ablated area of the sample was assessed quantitatively and compared with non-modified areas. The 3D image of the sample was segmented and arbitrary sections horizontal (in the plane of the sample) and vertical (perpendicular to the plane of the sample) were taken. In these sections, a Laplacian filter was applied to cuts of the area near the sample surface to determine the sample edge. The edge images were converted to 2D plots. The plots were smoothed by a Gaussian filter. As a measure of the roughness, the variations in the difference between the original curve and the smoothed one were taken into consideration for analysis. A total of 12 curves of four sections were processed to obtain the data shown in Figure 10.

Half of the curves were from non-ablated areas and the other half from ablated areas. The error bar represents the standard deviation of the mean variance of the six cuts in the corresponding group. As seen in Figure 10, the variation in the ablated areas was ~40 μm compared to ~8 μm for non-ablated areas. These results confirmed the improvement of the surface roughness after fs laser processing.

### 3.3. Changes in Crystallinity of PCL in Relation to Femtosecond Laser Treatment

**Figure 11:**
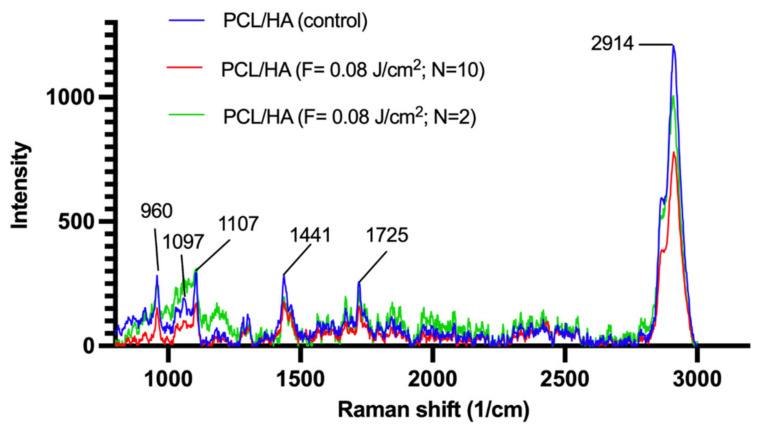
Raman spectra of PCL/HA scaffold before and after laser treatment: λ = 800 nm; F = 0.08 J/cm^2^; N = 2 and 10.

As mentioned earlier, PCL is a semicrystalline polymer, and its level of crystallinity could be affected by laser processing. The investigation of the level of changes in the 3D-printed scaffolds’ structure after the femtosecond laser treatment was performed via Raman spectroscopy (Figure 11). Peaks showing the samples crystallinity have been identified for the vibrational modes of skeletal stretch region (850–1110 cm^−1^; C–COO stretch and C–C stretch), CH_2_ twist (1270–1320 cm^−1^), CH_2_ bend (1405–1470 cm^−1^), C=O stretch (1710–1750 cm^−1^) and CH stretch (2800–3200 cm^−1^) [41,42]. The spectra of the samples before laser treatment showed peaks confirming the skeletal stretch region, CH_2_ bend, C=O stretch and CH stretch (Figure 11), which indicated the crystal phases within the material.

The research of Kister et al. and Baranowska-Korczyc et al. has shown that at 1720 cm^−1^, two peaks could be observed—one sharper, indicating the presence of a higher number of crystalline regions, and another smoother, indicating the presence of amorphous regions [42,43]. In the control sample, we could see an intense peak in this region, which became less pronounced after laser ablation. The increase in applied laser pulses led to a decrease in peak intensities and to broadening of the peaks for all of the identified spectral regions. It has been presented by Kister et al. that broader peaks are observed in the Raman spectra of amorphous PCL, but not in the one of semicrystalline PCL [43]. The main marker for the amorphous phase is a peak at ~865 cm^−1^, which was not detected in our results [42]. The findings of our work would lead us to the conclusion that (i) the melting temperature at 350 °C did not cause significant alterations in the structure of the polymer and it rather retained its semicrystalline state, and (ii) femtosecond laser treatment interacted with the crystal regions of the polymer, possibly leading to a transition from a crystalline to amorphous state. Additionally, according to literature, the highest peak of the hydroxyapatite Raman spectrum is detected at 960 cm^−1^ [44]. Due to overlapping with the PCL spectrum, at this point, we could not be fully certain of which material yielded higher peak intensity.

**Figure 12:**
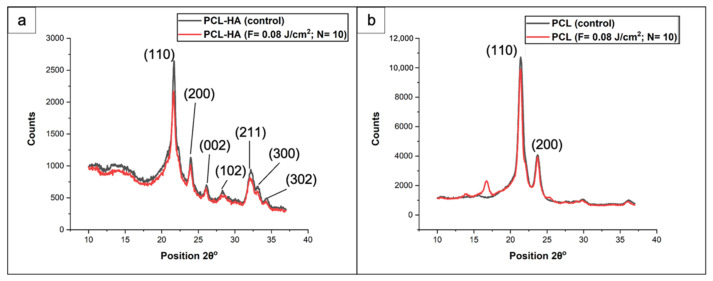
XRD spectra of control and femtosecond-treated PCL/HA (**a**) and PCL (**b**) scaffolds (λ = 800 nm; F = 0.08 J/cm^2^; N = 10).

Further information on the structural conformation of PCL and PCL/HA scaffolds was obtained from the performed XRD analysis (Figure 12).

The distinct peaks identifying PCL were detected in both types of scaffolds: 21.4° and 23.7°, corresponding to (110) and (200) planes, respectively. The presence of hydroxyapatite was confirmed by the characteristic peaks at 32.2° and 33°, correlated to the planes (211) and (300), respectively. Additional peaks at 26° (002 plane), 28.3° (102 plane) and 32.3° (202 plane) added more evidence on the preservation of the crystal structure of the apatite. These results confirmed findings in the literature [45,46]. When the data of untreated samples were compared with the laser-treated ones, there was no substantial difference—in the cases of both types of scaffolds, the signal intensity of the laser-ablated samples was slightly lower for all peaks. We could conclude from these findings that the femtosecond laser treatment did not significantly change the crystal structure of the materials under investigation.

### 3.4. Effects of Femtosecond Laser Treatment on PCL and PCL/HA Scaffold Wettability

**Figure 13:**
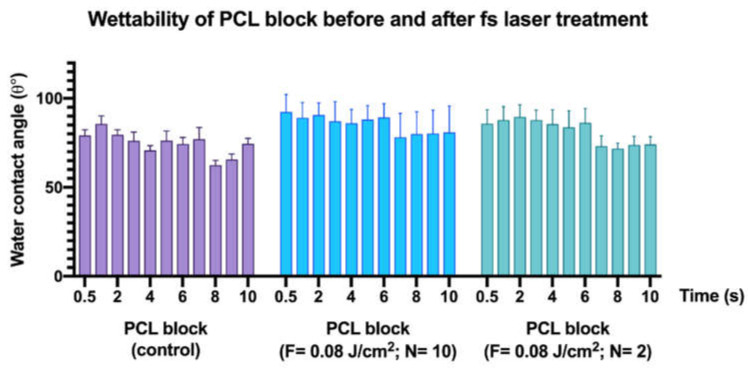
Wettability assessment of a 3D-printed PCL block, modified with fs laser: λ = 800 nm; F = 0.08 J/cm^2^; N = 10 and 2.

The water contact angle measurements were performed on the 3D-printed block of PCL modified under irradiation regime of λ = 800 nm, F = 0.08 J/cm^2^ and N = 10 and 2 (Figure 13).

**Figure 14:**
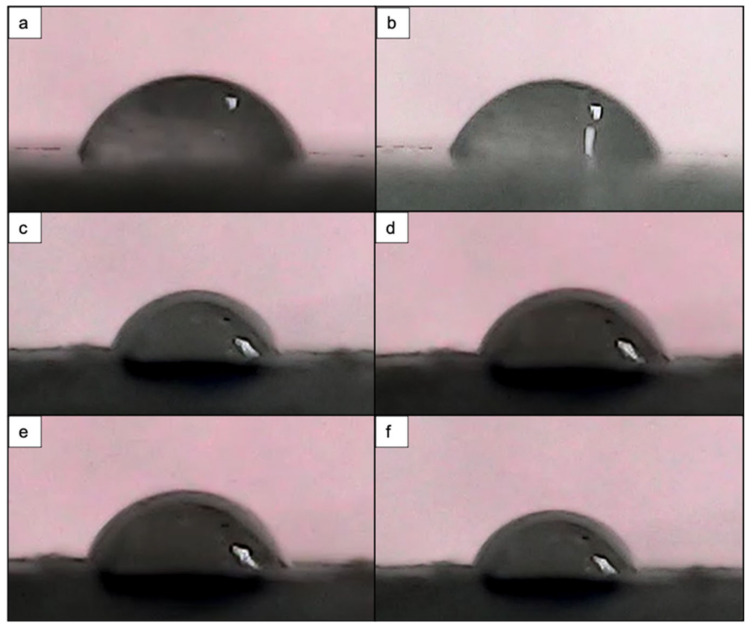
Water droplet evolution on control and fs laser-treated 3D-printed block of PCL. (**a**) Control area at 0.5 s with θ = 79°. (**b**) Control area at 10 s with θ = 74.5°. (**c**) Fs laser-treated area (λ = 800 nm; F = 0.08 J/cm^2^; N = 10) at 0.5 s with θ = 92.4°. (**d**) Fs laser-treated area (λ = 800 nm; F = 0.08 J/cm^2^; N = 10) at 10 s with θ = 81°. (**e**) Fs laser-treated area (λ = 800 nm; F = 0.08 J/cm^2^; N = 2) at 0.5 s with θ = 86°. (**f**) Fs laser-treated area (λ = 800 nm; F = 0.08 J/cm^2^; N = 2) at 10 s with θ = 74.2°.

Overall, the results of both laser-processed and control samples followed a similar behavior over the 10 s period of drop evolution, as the contact angle decreased (Figure 14). However, the change in WCA values did not follow a linear decrease, but rather fluctuated. These observations could be attributed to the fact that the water droplet deployed over the laser-textured areas had first interacted with the highest peaks of the formed microstructures, entrapping air between itself and the rough surface underneath [47]. Nevertheless, due to the irregular profile of the microstructures within the microchannels, the amount of entrapped air could vary, thus affecting the spreading of the water droplet over the modified area. Additionally, the fluctuations and the overall decrease in WCA values over the 10 s time period could potentially be explained by a transition between the Cassie–Baxter and Wenzel wetting states, both of which follow the contact of a drop with rough surfaces [48]. The statistical analysis (a two-way ANOVA) revealed a *p* = 0.9937 (*p* > 0.05); thus, the results were deemed statistically insignificant.

**Figure 15:**
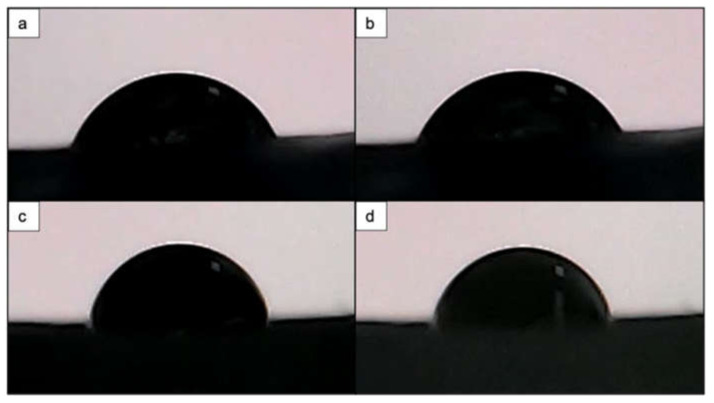
Water droplet evolution on control and fs laser-treated PCL scaffold. (**a**) Control area at 0.5 s with θ = 69°. (**b**) Control area at 10 s with θ = 72.3°. (**c**) Fs laser-treated area (λ = 800 nm; F = 0.08 J/cm^2^; N = 10; d_y_ = 32 μm) at 0.5 s with θ = 81°. (**d**) Fs laser-treated area (λ = 800 nm; F = 0.08 J/cm^2^; N = 10; d_y_ = 32 μm) at 10 s with θ = 73.2°.

Concerning the wettability of the PCL and PCL/HA scaffolds, the most prominent result was obtained for the PCL matrix with wood pile geometry, irradiated with F = 0.08 J/cm^2^, N = 10 and a d_y_ = 45 μm. It demonstrates a superhydrophilicity due to the observation of surface complete wetting. The Raman spectra of the modified samples showed a reduction in the peak intensity of the hydrophobic CH_2_ groups, which could possibly explain the increase in the WCA values. Contrary to this finding, the PCL scaffold treated with the same laser parameters but with a lower d_y_ (32 μm) showed a WCA of 73° which was, in fact, 2° higher than the control sample (Figure 15).

**Figure 16:**
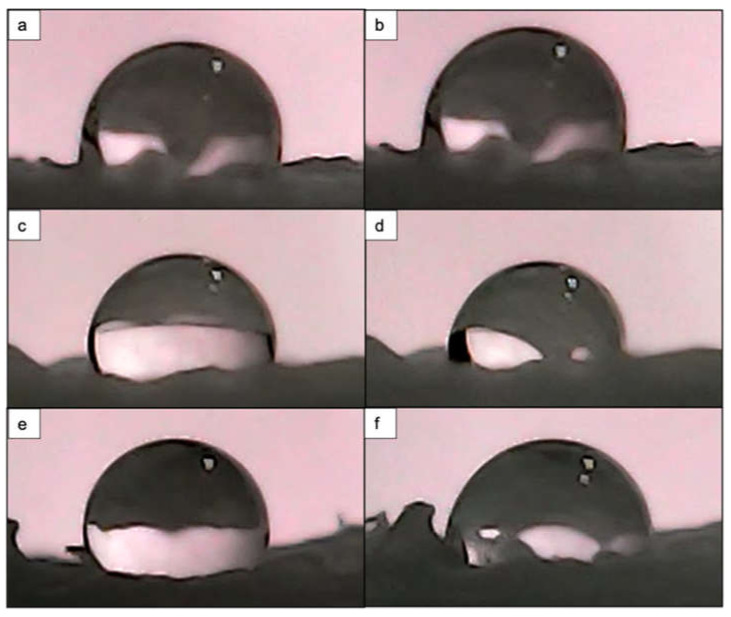
Water droplet evolution on control and fs laser-treated PCL/HA scaffold. (**a**) Control area at 0.5 s with θ = 89°. (**b**) Control area at 10 s with θ = 93.2°. (**c**) Fs laser-treated area (λ = 800 nm; F = 0.08 J/cm^2^; N = 10; d_y_ = 45 μm) at 0.5 s with θ = 101.3°. (**d**) Fs laser-treated area (λ = 800 nm; F = 0.08 J/cm^2^; N = 10; d_y_ = 45 μm) at 10 s with θ = 85°. (**e**) Fs laser-treated area (λ = 800 nm; F = 0.08 J/cm^2^; N = 2; d_y_ = 45 μm) at 0.5 s with θ = 101.7°. (**f**) Fs laser-treated area (λ = 800 nm; F = 0.08 J/cm^2^; N = 2; d_y_ = 45 μm) at 10 s with θ = 95.3°.

In the case of PCL/HA composites, the laser treatment did not lead to significantly improved wettability. For the period of 10 s, both laser-processed and control samples showed differences in WCA in the range of ~10° (Figure 16).

Similar results were shown in the research of Jiang et al. and Rajzer, who also observed a WCA above 90° for a PCL/HA composite fibrous scaffold, but with a lower concentration of hydroxyapatite within their composites [49,50]. In the work of Rajzer, it has been suggested that the presence of the minerals increased surface roughness, which in turn reduced the wettability of the material [50]. However, in our case, the comparison of surface roughness between control samples of pure PCL material and PCL/HA composite showed that the addition of the minerals did not substantially increase the roughness. The RMS value for control PCL was measured to be 8.98 μm, while that for the control PCL/HA composite was 4.48 μm. Further experiments with varying hydroxyapatite concentrations should be carried out to fully investigate the lower wettability of the samples after the addition of the minerals.

### 3.5. Evaluation of Microhardness before and after Femtosecond Laser Processing of PCL and PCL/HA Scaffolds

The results of the microhardness test performed are given in Table 2. All values, averaged over five separate measurements, are given in (HV).

We monitored a slight decrease in the average values in the fs laser-treated samples. However, Tukey’s multiple comparisons test showed no statistical significance between each of these groups. Only the comparison between the cPCL block samples with each of the rest was found statistically significant.

The slightly lower HV values of the 3D samples, compared to the control thick plate PCL, can be attributed to the mesh structure of the 3D-printed scaffolds.

## 4. Discussion

In our work, we fabricated PCL and PCL/HA scaffolds by 3D printing and subsequently processed them with a femtosecond laser in order to create surface patterns with distinct topography and roughness. We aimed at achieving functional surfaces that could reduce bacterial adhesion and promote human cell attachment and proliferation.

### 4.1. Laser Texturing and Structures Morphology

As mentioned in the Introduction Section, bacterial infections constitute a major risk in the post-implantation process. In our work, we found out that the femtosecond laser ablation with a fluence of 0.08 J/cm^2^ and N = 2 resulted in the development of 3D microcolumns on the PCL surface. Separate columns were seen on the PCL scaffold towards the edges of the pores, while on the PCL/HA composites, the protrusions had fused in parallel arrays (Figure 6b). Despite having single columns, the majority of them had merged, forming a continuous sheet. The swelling of the PCL expressed in formation of microcolumns could be explained with the nature of the ultra-short laser matter interaction that can lead to athermal phase transformations, direct bond-breaking and explosive disintegration. The reason for the microcolumns’ formation on the PCL fibers could be explained with the rapid heating and cooling of the material and ultrafast mechanical deformation, which eventually leads to this specific form of material ejection and the characteristic volume expansion in the form of bump-like structures. The combination of the applied laser parameters and the properties of the biodegradable polymer such as toughness and elasticity is the key for obtaining the specific morphology. The addition of HA makes the material stronger and harder, but more rigid, as well—the interaction with the fs laser beam ejects the material out of the contact zone, leading to formation of microprotrusions. After initial observation obtained with SEM analysis, we can hypothesize that the column-like morphology could be utilized as a strategy for bacterial entrapment in between the columns, hence restricting migration. However, to achieve this goal, further optimization of the experimental procedure with the same parameters will be required.

One approach for creating an antimicrobial surface is the development of a network of single 3D microstructures that could entrap bacteria by restricting their movement and interaction with adjacent microbes. Several studies have fabricated pillars at a micro- and nanolevel onto the surface of different materials. Varying heights and arrangements have yielded different roughness, but the results have shown that pillars ranging between 0.5 μm and 1.2 μm in height with the same range for distance in between single pillars have reduced bacterial adherence to the largest extent [18,51]. It is of key importance that the length between the structures is kept within 2 μm, so that it does not allow the aggregation of bacteria [18]. The morphology of the patterns is also crucial—for example, creating 3D micro- or nano-cylinders with wider diameter might not allow bacteria to move in between single motifs; however, the microbes could adhere on top of the cylinders as there would be a larger flat area for contact [52]. For example, creating micro spikes could lead to reduced points of contact; thus, bacteria would not be able to adhere completely [53].

When an implant is placed at the site of injury, apart from hindering bacterial attachment, it should also promote human cell attachment. Hence, the scaffold would better integrate into the organism, and it would stimulate the injured tissue regeneration. For the scaffold fabrication, we chose two types of geometries—a wood pile one and a snowflake-like one. These were chosen based on evidence from the literature that triangular pores formed from the crossing of the fibers during the 3D printing process have a beneficial impact on cell attachment and proliferation [54]. The researchers studied both the shape and the size of the pores and observed that in 0.5 mm triangular pores, undifferentiated placenta-derived cells could differentiate towards the osteogenic lineage, even without lineage-promoting factors. Van Bael et al. concluded that the combination of smaller pores, which have a better impact on the initial cell adhesion, and larger pores (1 mm), which prevent occlusion of the pores while still promoting cell viability, would strongly improve the integration of the scaffold within the body [54]. In order to exert an additional effect on the migration and orientation of cells, we utilized femtosecond laser treatment for the pattering of PCL and PCL/HA surfaces. By applying 10 laser pulses per spot, we observed the formation of microchannels which could have guiding functions for the cells during their migration. From these results, we assume that despite the irregularities and the zones with partially fused material, such microchannels could potentially have an impact on cell guidance and motility control [55]. Moreover, the pores that have formed within the channels could further stimulate the cellular integration within the scaffold, hence aiding the process of regeneration [38].

Holthaus et al. studied how different widths of microchannels affected the proliferation and orientation of osteoblasts [39]. The authors found that the highest number of cells grew in parallel in channels with diameters between 40 and 80 μm; however, still some cells grew on top of the channels. Based on these results, we could hypothesize that future in vitro studies with bone cells seeded on the channels created in the PCL/HA scaffold with an average diameter of 43.2 μm should yield suitable results for enhancement of cellular orientation and adhesion. The morphological analyses of laser-treated PCL and PCL/HA showed that a better porosity was present in the pure material, while improved roughness was seen in the composite scaffold. These two factors are crucial for cell attachment and infiltration as well as for proper nutrient and waste diffusion [56]. The optimal pore size has been debated as different studies indicate various results. A general conclusion has been made that pores with size above 300 μm are promoting bone ingrowth and vascularization of the scaffold, while pores below 300 μm stimulate the formation of bone–cartilage interphase [56]. The combination of the pores arising from the inherent porosity of the PCL as a material with an average diameter of 11 μm and the ones arising from the geometry of the scaffold as a result of the fabrication process—700 μm (wood pile) and 800 μm (snowflake-like) could improve both the cellular infiltration within the scaffold and the native bone tissue ingrowth. The smaller micropores would allow the diffusion of nutrients through the material, further promoting the tissue regeneration process.

### 4.2. Chemical Composition

In the current study, our second goal was to design a composite matrix with an addition of hydroxyapatite in order to promote the osteointegration of the scaffold in the body and to stimulate in that way the formation of a new bone tissue. The performed morphological and EDX analyses confirmed the homogeneous distribution of the minerals within the scaffold. The EDX analysis revealed partial ablation of PCL material, which led to the acquisition of higher quantities of Ca and P elements. The laser–material interaction had caused clustering of minerals, thus increasing the weight % of the two minerals in the laser-modified areas. The inorganic particles were detected at the surface of the composite scaffold in both laser-treated and control areas, which is very important for the initial contact of the bone cells with the material.

### 4.3. Wettability Evolution and Mechanical Stability

Surface wettability is one of the properties that must be considered when monitoring the interaction between cells and bacteria with surfaces [8]. Since PCL generally exhibits hydrophobic properties, we performed water contact angle analysis to assess how the femtosecond laser treatment shifted the degree of wettability. For this purpose, we performed the analysis on both a laser-treated 3D-printed solid block of PCL and on the two types of 3D-printed scaffolds (PCL and PCL/HA). The obtained results showed that the contact angle on the control areas decreased in comparison to the laser-modified areas. Nevertheless, for a 10 s period of drop evolution, we observed a slight improvement in wettability in the laser-textured areas (Figure 13). Different observations were noted when the analysis was performed on the PCL scaffold with wood pile geometry, where the application of N = 10 (F = 0.08 J/cm^2^; d_y_ = 45 μm) led to complete wetting of the surface, while the control area retained a contact angle of 71°. Similar values for the contact angle of the control sample were also measured on the same type of 3D-printed PCL scaffold with crossing fibers by Kosik-Kosiol et al. and Wibowo et al. [57,58]. The achieved wettability under the above-mentioned treatment tuned the PCL surface properties to more hydrophilic ones, which has been investigated to have a negative impact on microbial attachment, but could be suitable for human cell adhesion and proliferation [59,60].

Another physical characteristic of the 3D-printed scaffolds that was evaluated was their hardness—the ability of a material to resist deformation. We compared the microhardness of the different groups of scaffolds and investigated whether the fs laser treatment (F = 0.08 J/cm^2^; N = 10) could alter this characteristic of the materials. The results of the indentation test (Table 2) concerning the PCL and PCL/HA mesh scaffolds indicated a slight decrease in the microhardness of fs laser-treated scaffolds when compared to control samples. In semicrystalline polymers, the microhardness is highly affected by the level of crystallinity. A higher crystallinity degree would result in improved microhardness [61]. Based on our results from Raman spectroscopy (Figure 11), we hypothesized that the fs laser irradiation led to a slight shift towards an amorphous state of the PCL, which could mean that the microhardness of the processed scaffolds could also decrease. Despite observing similar trends in between the different analyses, still the correlations between fs laser-treated groups and their corresponding control ones in the Vickers microhardness test were statistically insignificant. Thus, we could not firmly conclude that the fs laser surface treatment led to a decrease in the microhardness of the scaffolds. However, we can conclude that from the obtained results in Table 2, the 3D-printed scaffolds with a wood pile-like or a snowflake-like geometry could potentially be used for regeneration of bone tissue injuries at non-load-bearing sites. For example, Vickers microhardness tests on human tibial diaphysis showed results ranging between 36.50 HV and 65.4 HV [62]. These values were, on average, 4.5× times higher than the values we obtained for PCL and PCL/HA scaffolds (Table 2). The combination of grooves having a rough morphology and increased porosity with the presence of hydroxyapatite could provide bone cells with optimal conditions for attachment, proliferation and for the synthesis of a new bone tissue [63]. Further experiments are foreseen to optimize the fabrication of composites with a higher concentration of hydroxyapatite, the addition of hydroxyapatite nanoparticles and a change in the laser ablation parameters (e.g., d_y_).

## 5. Conclusions

In our work, we investigated the influence of femtosecond laser processing on the formation of stripe-like patterns on the surfaces of 3D-printed PCL and PCL/HA scaffolds. The acquired surface designs on the individual struts of PCL and PCL/HA matrices in the form of stripes and micro columns represent a model for the creation of cell-friendly and bacteria-repellent scaffolds. Post-ablation alterations were observed not only in the surface morphology of the materials, but also in their physical properties, namely, the level of wettability. We hypothesize that our results, specifically the formation of microchannels that led to complete wetting of the modified PCL surface, could be the basis required for the next step in developing implants combining antibacterial and osteoinductive properties.

The future steps of our experimental methodology will emphasize on the effects of the surface modifications described in this article on cells and bacteria behavior. In particular, the microchannels formed on PCL and PCL/HA scaffolds will be studied for their potential effects on human bone cell proliferation, guidance and differentiation. The promising influence of the obtained micro column formations on bacterial attachment and motility restriction will be examined in experiments with Gram-positive (*S. aureus*) and Gram-negative bacteria (*E. coli*).

## Figures and Tables

**Table 1:** Quantitative analysis of elements within a PCL/HA and PCL scaffold before and after femtosecond laser ablation (λ = 800 nm; F = 0.04 J/cm^2^; N = 10).

Element	PCL/HA Scaffold Weight (%)		PCL Scaffold Weight (%)	
	Control	Ablated	Control	Ablated
C	49.71	57.88	50.92	72.42
O	47.28	22.59	49.98	27.58
Ca	2.21	13.13	N/A	N/A
P	0.80	6.40	N/A	N/A

**Table 2:** Vickers microhardness test results in (HV).

№	cPCL- Block	3D cPCL	3D fsPCL	3D cPCL + HA	3D fsPCL + HA
1	23.0	12.4	11.1	12.1	8.1
2	16.9	11.9	9.8	12.6	11.4
3	18.3	12.5	12.3	11.4	12.5
4	21.1	13.0	13.1	13.3	10.7
5	14.8	11.1	10.9	9.9	9.8
Averaged value	18.82	12.18	11.44	11.86	10.5

## Data Availability

Data available in a publicly accessible repository.
